# Influence of Mild Chronic Stress and Social Isolation on Acute Ozone-Induced Alterations in Stress Biomarkers and Brain-Region-Specific Gene Expression in Male Wistar–Kyoto Rats

**DOI:** 10.3390/antiox12111964

**Published:** 2023-11-03

**Authors:** Matthew C. Valdez, Danielle L. Freeborn, Joseph M. Valdez, Andres R. Henriquez, Samantha J. Snow, Thomas W. Jackson, Prasada Rao S. Kodavanti, Urmila P. Kodavanti

**Affiliations:** 1 Neurological and Endocrine Toxicology Branch, Public Health and Integrated Toxicology Division, CPHEA/ORD, US Environmental Protection Agency, Research Triangle Park, NC 27711, USA; valdez.matthew@epa.gov (M.C.V.); freeborn.danielle@epa.gov (D.L.F.); valdez.joseph@epa.gov (J.M.V.); kodavanti.prasada@epa.gov (P.R.S.K.); 2 Oak Ridge Institute for Science and Education Research Participation Program, US Department of Energy, Oak Ridge, TN 37831, USA; andhencor@gmail.com (A.R.H.); jackson.thomas@epa.gov (T.W.J.); 3 Cardiopulmonary and Immunotoxicology Branch, Public Health and Integrated Toxicology Division, CPHEA/ORD, US Environmental Protection Agency, Research Triangle Park, NC 27711, USA; samantha.snow@icf.com

**Keywords:** hypothalamus, hippocampus, bed nucleus of the stria terminalis, olfactory bulbs, gene expression, metabolomics, glucocorticoids, *Fkbp5*, chronic stress, social isolation, neuroendocrine, ozone

## Abstract

Individuals with psychosocial stress often experience an exaggerated response to air pollutants. Ozone (O_3_) exposure has been associated with the activation of the neuroendocrine stress-response system. We hypothesized that preexistent mild chronic stress plus social isolation (CS), or social isolation (SI) alone, would exacerbate the acute effects of O_3_ exposure on the circulating adrenal-derived stress hormones, and the expression of the genes regulating glucocorticoid stress signaling via an altered stress adaptation in a brain-region-specific manner. Male Wistar–Kyoto rats (5 weeks old) were socially isolated, plus were subjected to either CS (noise, confinement, fear, uncomfortable living, hectic activity, and single housing), SI (single housing only, restricted handling and no enrichment) or no stress (NS; double housing, frequent handling and enrichment provided) for 8 weeks. The rats were then exposed to either air or O_3_ (0.8 ppm for 4 h), and the samples were collected immediately after. The indicators of sympathetic and hypothalamic–pituitary axis (HPA) activation (i.e., epinephrine, corticosterone, and lymphopenia) increased with O_3_ exposure, but there were no effects from CS or SI, except for the depletion of serum BDNF. CS and SI revealed small changes in brain-region-specific glucocorticoid-signaling-associated markers of gene expression in the air-exposed rats (hypothalamic *Nr3c1*, *Nr3c2 Hsp90aa1*, *Hspa4* and *Cnr1* inhibition in SI; hippocampal *HSP90aa1* increase in SI; and inhibition of the bed nucleus of the stria terminalis (BNST) *Cnr1* in CS). Gene expression across all brain regions was altered by O_3_, reflective of glucocorticoid signaling effects, such as *Fkbp5* in NS, CS and SI. The SI effects on *Fkbp5* were greatest for SI in BNST. O_3_ increased *Cnr2* expression in the hypothalamus and olfactory bulbs of the NS and SI groups. O_3,_ in all stress conditions, generally inhibited the expression of *Nr3c1* in all brain regions, *Nr3c2* in the hippocampus and hypothalamus and *Bdnf* in the hippocampus. SI, in general, showed slightly greater O_3_-induced changes when compared to NS and CS. Serum metabolomics revealed increased sphingomyelins in the air-exposed SI and O_3_-exposed NS, with underlying SI dampening some of the O_3_-induced changes. These results suggest a potential link between preexistent SI and acute O_3_-induced increases in the circulating adrenal-derived stress hormones and brain-region-specific gene expression changes in glucocorticoid signaling, which may partly underlie the stress dynamic in those with long-term SI.

## 1. Introduction

Air pollution is the fourth highest risk factor for human mortality worldwide, contributing to nearly 70% of all environmental causes of deaths [1]. In developing communities, where rapid urbanization and industrialization are coupled with overpopulation and socioeconomic disparities, the mortalities caused by air pollutants are more prominent [1]. Recent evidence has shown that exposure to air pollutants affects all organ systems, and it is especially associated with adverse neurobehavioral outcomes and chronic neural and systemic diseases [2,3,4].

Exposures to particulate and/or gaseous air pollutants have been associated with several neuropsychological ailments, including an increased incidence of Alzheimer’s disease [5], Parkinson’s diseases [4], cognitive decline [6], depression [7], mood disorders [8], decreased hippocampal volume [9] and increases in stress biomarkers [10]. The epidemiological evidence shows that there is an interactive influence between socioeconomic stressors and air pollutants in the exacerbation of adverse health outcomes for neural and systemic chronic diseases [11,12]. An increased allostatic load, which is generally defined as the cumulative, adverse health outcomes of a life-long exposure to chemical and non-chemical stressors on the stress-response system and the body, has been associated with both air pollution and lower socioeconomic status [13].

Experimentally, the acute and long-term exposure to air pollutants have caused neuroinflammation [14], oxidative and metabolic alterations [15,16], the exacerbation of Alzheimer’s disease phenotypes [17,18], behavioral anomalies [19] and, more importantly, the activation of the sympathetic–adrenal medullary (SAM) and the hypothalamic–pituitary–adrenal (HPA) axes in rodents [20,21]. In many cases, these neural changes are observed from a gaseous air pollutant, such as ozone (O_3_), which is not likely to translocate to the brain upon inhalation [22]. While the SAM and HPA axes, induced by stress through the release of catecholamines and glucocorticoids, have also been implicated in a variety of chronic, psychiatric stress conditions [23,24], it is conceivable that there are likely interactive effects from multiple chemical and non-chemical stressors in the mediation of the neural and neuropsychiatric health effects of air pollutants. An impairment in the responsiveness of the neuroendocrine system to external or physiological stress has been implicated in neural and chronic systemic diseases [23,24]. There is emerging evidence that O_3_ activates several stress-responsive regions across the central nervous system (CNS) [25], and we have shown that the neuroendocrine stress response induced by O_3_ underlies the neural, pulmonary, and systemic effects through SAM and HPA activation, and the subsequent release of catecholamines and glucocorticoids [26,27]. In addition to neuroendocrine activation, acute high concentrations of O_3_ exposures have been shown to induce oxidative stress, alter antioxidant homeostasis and activate microglial cells, while long-term exposure at lower concentrations has been shown to reduce synaptic plasticity and increase the oxidative burden [28,29,30]. These diverse effects of acute O_3_ exposure in various brain regions, in addition to increasing the circulating adrenal-derived hormones, might also influence the effectiveness of the central glucocorticoid stress response to O_3_.

In this study, we hypothesized that different preexistent long-term stress conditions would modify sensitivity to O_3_-induced glucocorticoid and catecholamine release, through transcriptional changes in their signaling pathways in the stress-responsive brain regions. These conditions were compared to a no-stress condition, which included the double housing of rats with environmental enrichment in cages and the frequent handling of rats on a daily basis. Male Wistar–Kyoto rats were selected for this study due to their reported depression phenotype, hypersensitivity towards stressors and exacerbated neuroendocrine responses [31,32,33]. Moreover, these rats have been extensively used in O_3_-inhalation toxicology studies involving neuroendocrine mechanisms [26,27]. Because catecholamines and corticosteroids feedback regulations have been implicated in the psychosocial stress-mediated mental health crisis, as well as resulting in systemic chronic diseases [23,24], and because acute O_3_ exposure increases these hormones, we presumed that a preexistent stress condition would impair the ability of O_3_ to mediate the signaling that leads to the activation of these neuroendocrine axes. Based on our prior study [34], acute O_3_ effects were assessed in a control, a socially isolated group and those exposed to chronic stresses, including social isolation. In addition to the lymphocytes, catecholamines and glucocorticoids that are typically related to HPA activation, the serum brain-derived neurotrophic factor (BNDF) was also analyzed. Furthermore, the gene expression levels for the markers of the glucocorticoid receptor system, glucocorticoid-signaling-related chaperone proteins, catecholamine signaling, and other related neurotrophic factors were analyzed to understand the potential interactive influence on adrenergic, glucocorticoid and cannabinoid signaling at transcriptional levels. The effects on the olfactory bulbs of the limbic regions involved in stress (hippocampus and hypothalamus) and the bed nucleus of the stria terminalis (BNST) [35] were selected to determine the brain-region-specific regulation of the stressor’s impact.

## 2. Materials and Methods

### 2.1. Animals

Male Wistar–Kyoto (WKY) rats (4-week-old) were purchased from Charles River Laboratories, Inc. (Raleigh, NC, USA). During a 1-week acclimation, animals were double-housed in cages with hardwood chip and enrichment material. The animal rooms were maintained at 21 °C, 55–65% relative humidity and a 12 h light/dark cycle. The EPA animal facility is approved by the Association for Assessment and Accreditation of Laboratory Animal Care. Throughout acclimation and the experimentation period, animals were fed Purina 5001 rat chow (Brentwood, MO, USA) and drank tap water, ad libitum, except during O_3_ or air exposure. The in-house Excel macro was used to randomize rats based on their body weights such that mean body weights in each treatment group remained similar. U.S. The EPA’s Institutional Animal Care and Use Committee approved the experimental protocol and we followed the guidelines of the National Institutes of Health for the care and use of rats [36].

### 2.2. Experimental Manipulations (Different Stress Conditions and O_3_ Exposure)

As reported in detail [34], five-week-old male WKY rats were subjected to no stress (NS), chronic mild stress (CS) or social isolation (SI) protocols in parallel (*n* = 24/group). The stress protocols were applied 42–44 times over 8 weeks until the day prior to O_3_ exposure. Some of the data on pulmonary injury/inflammation and serum clinical stress markers have been published recently [34].

#### 2.2.1. No Stress (Control; NS)

Rats in the NS group were double-housed and provided EnviroDry (crinkled paper) as nesting material and as environmental enrichment. These animals were handled for weighing and different testing protocols (Table 1).

#### 2.2.2. Social Isolation Only (SI)

For the SI group, animals were single-housed with no environmental enrichment added to the cage, and handling was kept to a minimum, such as weekly weighing and cage changes. This was designed to develop the SI phenotype. We presumed that WKY rats, due to noted preexistent depressive phenotypes in this strain attributed to their abnormalities in cholinergic mechanisms in the brain [37], would serve to represent some of the characteristics of human social isolation (Table 1).

#### 2.2.3. Unpredicted Chronic Mild Stress plus Social Isolation (CS)

In order to induce a more complex stress phenotype, rats in one group were socially isolated by single housing without enrichment plus application of varied mild unpredictable randomized stressors (Table 1). This group is called CS, and it contrasts with the SI group of rats that were socially isolated but did not undergo the variable stress paradigm. Rats were subjected to one of the 5 stressors listed in Table 1 (restraint, shaking, tilted cage, exposure to predator odor or noise). All stressors were applied for 1 h/day except for noise, which was applied for 6 h/day. Each stressor was applied in a random manner over 5 days per week for 8 weeks, Monday through Friday. The stressor application continued during the weekend after the 8th week, until the day prior to O_3_ exposure. This stress condition was applied in rats that were single-housed and provided no enrichment material in the cages. The order in which these stressors were applied was randomized to decrease the chance for habituation to a given individual stressor.

#### 2.2.4. O_3_ Generation and Exposure

On the 9th week of NS, CS or SI, animals were exposed to filtered air or O_3_ [22,34]. A silent arc discharge generator (OREC, Phoenix, AZ, USA) generated O_3_ from oxygen. Mass flow controllers were used to regulate the entry of O_3_ into the Rochester-style “Hinners” chambers. Photometric O_3_ analyzers (API Model 400) were used to monitor the O_3_ concentrations in the chambers. Rats were exposed to filtered air or O_3_ (0.8 ppm) for 4 h. Exposure chamber conditions and actual O_3_ concentrations were monitored during each exposure and reported previously [22,34]. The O_3_ concentration used in the present study is an order of magnitude higher than what is encountered in non-attainment areas in the US [38]; however, this concentration in resting rats is comparable to the levels used in human clinical studies conducted during intermittent exercise based on airway dose deposition [39]. Ambient O_3_ concentrations around 0.4 ppm have been reported in tropical countries [40]. During O_3_ exposure, rats were individually housed in wire-mesh cages. Food and water were not available during exposures.

### 2.3. Tissue Sample Collection and Processing

On the day following the final stressor application of NS, CS and SI, rats were exposed to filtered air or O_3_ (0.8 ppm) from 7 am to 11 am, followed by tissue collection within 2 h (11 am–1 pm). During tissue collection, animals were staggered such that one animal from each treatment group was processed first, followed by the subsequent animal from each group, to reduce potential sample error between treatment groups. Rats were euthanized with an overdose of sodium pentobarbital (Virbac AH, Inc., Fort Worth, TX, USA; >200 mg/kg, i.p.). Blood samples were collected through the abdominal aorta directly into the vacutainer EDTA blood tubes and serum separator tubes (generally 3–5 min after administering pentobarbital). Brains were removed, and brain regions (olfactory bulbs, hypothalamus, hippocampus and BNST) were dissected on ice, flash-frozen on dry ice and stored at −80 °C until analyzed.

#### 2.3.1. Serum/Plasma Biomarkers

Hematological parameters and white blood cell differentials were investigated using EDTA blood tubes on a Beckman-Coulter AcT blood analyzer (Beckman-Coulter Inc., Fullerton, CA, USA). EDTA and serum separator tubes were centrifuged (3500× *g* for 10 min) and plasma/serum samples were aliquoted and stored at −80 °C. Plasma levels of corticosterone, epinephrine and nor-epinephrine were analyzed using Rocky Mountain Diagnostics Inc ELISA kits (Colorado Springs, CO, USA) following the kit protocol. A SpectraMax i3x Multi-Mode Microplate Reader (Molecular Devices, San Jose, CA, USA) was used for reading ELISA plates. Serum levels for adrenocorticotropic hormone (ACTH), pituitary hormone and brain-derived natriuretic factor (BDNF) were analyzed using a MILLIPLEX MAP Rat Pituitary Magnetic Bead Panel (Merck-Millipore, Burlington, MA, USA) kit, and plates were read on a Luminex 200 System (Millipore Sigma, Burlington, MA, USA). The data for other hormones have been published previously [34].

#### 2.3.2. Tissue Processing, RNA Isolation and Quantitative Polymerase Chain Reaction

Snap-frozen brain regions were used to isolate RNA via a spin-column-based isolation kit (Qiagen RNeasy Mini Kit #74104). Samples were homogenized with Omni international tissue homogenizer (Omni International, Kennesaw, GA, USA; #59136) in TriZol (Thermo Fisher, Waltham, MA, USA; #15596026), then precipitated with chloroform (Sigma-Aldrich, St.Louis, MO, USA; C5312). Ethanol (70%) was added to the aqueous phase before being processed through the RNeasy spin column. The amount of RNA per sample was quantified using the Qubit 4 Fluorometer (Invitrogen, Waltham, MA, USA; Q33238) and Qubit RNA BR Assay Kit (Invitrogen, Q10211) and then diluted to 1 ng/µL with nuclease-free water (Ambion, Austin, TX, USA; AM9938). The PCR was run using the Bioline SensiFAST™ SYBR^®^ No-ROX One-Step Kit (Bioline, Thomas Scientific, Swedesboro, NJ, USA; BIO-72005). Primers were designed in-house and synthesized by Integrated DNA Technologies (Table 2). Efficiency curves were constructed for all primers, and only primer sets with efficiencies of 90–110% were accepted for use in this study. All PCRs were performed on the Quant Studio 7 Flex PCR Machine. All PCR reactions underwent melt curve analysis to verify that only single amplicons were amplified. For analysis, an R script was written to perform the Pfaffl equation for relative fold gene expression using b-*Actin* as the normalization transcript [41]. In brief, the gene expression ratio is equal to the quotient of the empirically determined efficiency of the gene of interest (GOI) to the power of the change in GOI Ct over the efficiency of the housekeeper gene (HK) to the power of the change in HK Ct. Prior to final analysis, three potential housekeeper genes (*Rpl13A*, *Gapdh* and b-*Actin*) were assayed and scrutinized to find the most stable reference gene for use in this study. Raw C_t_ values for b-*Actin* led us to determine it was the least variable reference gene across groups and, therefore, represented the most stable housekeeping gene (for example, for the NS group, the raw Ct values for air/O_3_ were: BNST, 19.82 ± 0.35/19.83 ± 0.36; hippocampus, 20.95 ± 0.41/20.65 ± 0.64; hypothalamus, 20.43 ± 0.36/20.54 ± 0.34; and olfactory bulbs, 20.08 ± 1.04/21.06 ± 1.77). Information on the primers used for the housekeeping genes and genes of interest is detailed in Table 2.

#### 2.3.3. Serum Metabolomics Analysis

Because preliminary results revealed that SI caused greater systemic effects than CS [22], serum global metabolomics was also performed in air- and O_3_-exposed animals from SI. Rat serum metabolomics was performed as described previously [42]. Data for NS condition air- and O_3_-exposed animals were recently published [42], and data for SI condition air and O_3_ exposure are presented herein. Briefly, one hundred microliters of sample were used for each analysis. Samples were prepared using the automated MicroLab STAR^®^ system from Hamilton Company. Internal standards specific to each chromatographic method were also added to the extract. The dried sample extract aliquots were reconstituted in solvents compatible with each of four LC-MS/MS methods using a series of isotopically labeled standards at fixed concentrations, to monitor injection and chromatographic consistency and to align chromatograms. In total, 1002 metabolites were detected with 887 of known identity. Peaks were quantified using the trapezoidal method of area under the curve calculation. Prior to statistical analysis, each compound was corrected in run-day blocks by registering the medians to equal 1.00 and normalizing each data point proportionately (termed the “block correction”). Following normalization, any missing values were imputed with sample set minimums on a per biochemical basis. An average of 46.5 metabolites were imputed for each sample, with 30 metabolites excluded from subsequent statistical analysis because >50% of samples were imputed. Each metabolite was analyzed in a two-way ANOVA (O_3_, stress) followed by specific contrasts within each fixed factor. For each metabolite, knowledge-based pathway annotations from the metabolomics platform were used (Metabolon, Inc., Morrisville, NC, USA).

### 2.4. Statistical Analysis

Statistical analyses were performed using RStudio [43]. Raw data were organized with the dplyr package [44]. All serum biomarker and PCR data were curated into one large comma-separated value (CSV) file. The dplyr package was used to group, filter and mutate (perform column statistics and biomarker/gene expression) the data. Two-way analysis of variance was used to analyze differences in biomarkers and gene expression using the inhalant exposure (O_3_ and air) and stressor condition (NS, CS, SI) as independent variables. Significant effects and/or interactions were assessed with two forms of post- hoc tests, of the main effect differences of exposure and within a stress condition. The R package, emmeans [45], was used to calculate the estimated marginal means, which are the mean responses for each factor adjusted for any other variables in the model, after a significant ANOVA result. To analyze average effects of CS and SI within each exposure group, Dunnett’s many-to-one multiple comparison test was performed using the trt.vs.ctrl contrast argument of the emmeans package. To analyze the effects of O_3_ within each stress condition, we performed pairwise comparisons using the pairwise contrast argument of emmeans. All figures were constructed with the ggplot2 package [46] and composite figures were plotted with the cowplot package [47].

## 3. Results

### 3.1. Phenotype Measures of the Stress Response

**Figure 1:**
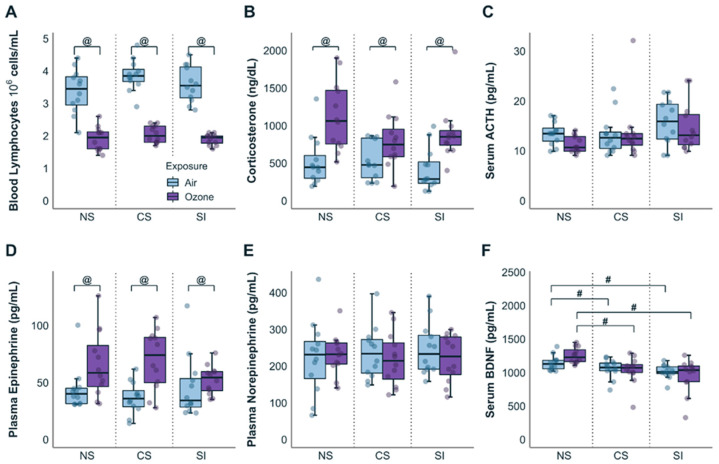
Effect of stressors on physiological responses. (**A**): Regardless of stress conditions, O_3_ decreased blood lymphocytes across all stress groups. (**B**): Corticosterone (CORT) was increased across all stress groups concomitant with the decrease in lymphocytes. (**C**): ACTH levels at the selected time point were unaltered across all stress and exposure groups. (**D**,**E**): Plasma epinephrine was significantly elevated by exposure to O_3_ with no effect on plasma norepinephrine levels. (**F**): BDNF plasma levels were significantly decreased by chronic stress and social isolation regardless of exposures. NS = no stress controls; CS = chronic stress group; SI = social isolation group. Data are represented by boxplots with a dot plot overlay to express the distribution of each data point where *n* = 5–12. @ represents a statistical difference (*p* < 0.05; see details in the text) between exposure groups within the same stress group. # represents a statistical difference (*p* < 0.05; see details in the text) between stress groups within the same exposure group.

Phenotypic markers of O_3_-induced stress effects and the influence of preexistent CS and SI were examined in plasma/serum samples. These data showed that no changes in circulating lymphocytes were noted in CS or SI groups exposed to clean air relative to the NS air group. Regardless of stress conditions, O_3_ lowered blood lymphocytes (F_(1,68)_ = 241.3; *p* < 0.0001) across all groups (Figure 1A). Corticosterone (CORT) was higher across all O_3_-exposed groups relative to air groups regardless of the underlying stress condition (Figure 1B; F_(1,68)_ = 28.82; *p* < 0.0001) concomitant with the decrease in lymphocytes. There were no significant changes in adrenocorticotropic hormone (ACTH) levels across stress and O_3_ groups (Figure 1C).

O_3_ exposure increases circulating epinephrine in healthy WKY rats [48,49], and the effects of CS and SI were examined on plasma catecholamines after an air or O_3_ exposure. Air-exposed CS and SI rats had similar levels of plasma epinephrine when compared to the air-exposed NS group. Plasma epinephrine (Figure 1D) was significantly elevated by O_3_ exposure (F_(1,68)_ = 14.84; *p* < 0.0005), with no effect on plasma norepinephrine levels (F_(1,68)_ = 0.4498; *p* = 0.5047) (Figure 1D,E). We determined the influence of CS and SI, as well as acute O_3_ exposure, on serum BDNF. BDNF levels were significantly (F_(2,68)_ = 10.2; *p* < 0.05) lower in CS and SI when compared to NS, regardless of air or O_3_ exposure (Figure 1F; SI < CS < NS). No acute O_3_ effects were observed on BDNF levels.

### 3.2. Gene Expression of Stress-Related HPA Activation

**Figure 2:**
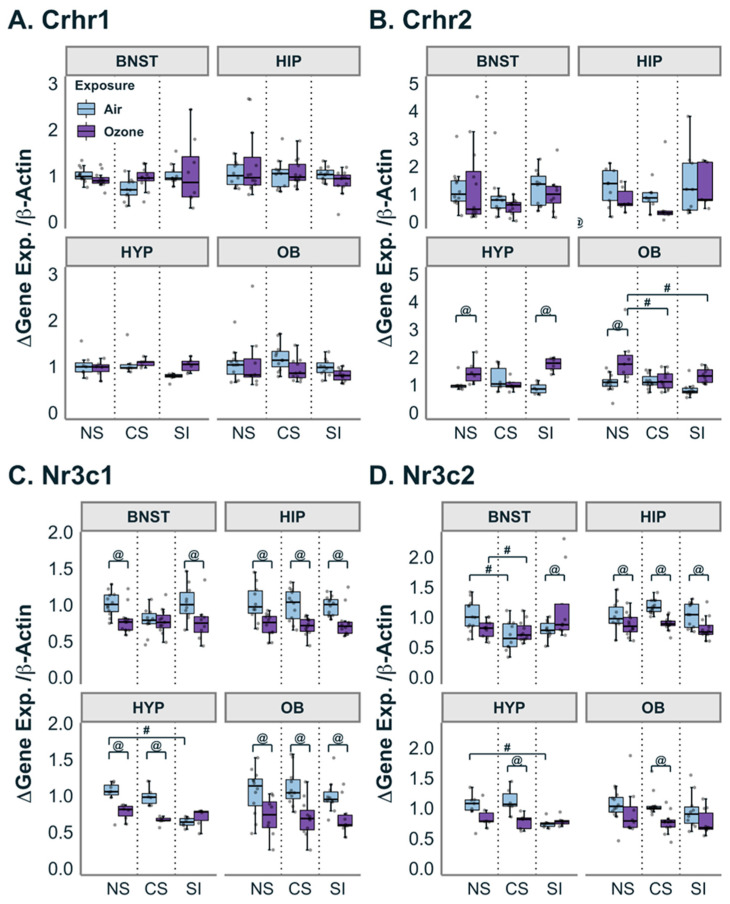
Effect of stressors on genes related to stress receptors. (**A**): Across all brain regions and stress groups, there was no change in *Crhr1* gene expression. (**B**): *Crhr2* expression was altered by O_3_ exposure in the no stress (NS) and social isolation (SI) group for hypothalamus (HYP) and NS group for olfactory bulb (OB). Also, in the OB, both chronic stress (CS) and SI had diminished responses to O_3_ exposure. (**C**): *Nr3C1* expression was significantly decreased by O_3_ exposure across all stress groups in the hippocampus (HIP) and OB. *Nr3C1* expression was also decreased in the NS and SI groups in the bed nucleus of the stria terminalis (BNST) and the NS and CS groups in the HYP. In the HYP, expression in the SI group was lower than that in the NS group. (**D**): *Nr3c2* expression was decreased by O_3_ for all three stress groups in the HIP. A similar decrease was observed in the HYP and OB, but only the CS groups were significantly decreased. In the BNST, O_3_ exposure in the SI group increased expression of *Nr3c2.* In the BNST and HYP, CS and S groups had diminished expression compared to NS, respectively. Data are represented by boxplots with a dot plot overlay to express the distribution of each data point where *n* = 5–12. @ represents a statistical difference (*p* < 0.05; see details in the text) between exposure groups within the same stress group. # represents a statistical difference (*p* < 0.05; see details in the text) between stress groups within the same exposure group.

To further investigate the role of the CNS in response to acute O_3_ and various forms of chronic stressors, we assayed gene expression from four brain regions: olfactory bulbs, BNST, hypothalamus and hippocampus. The expression of corticotropin-releasing hormone (CRH) receptor *Crhr1* was not changed by either O_3_ exposure or any stress conditions (CS or SI) in any brain regions examined (Figure 2A). *Crhr2* expression was not altered by CS or SI in any brain regions of air-exposed animals relative to air-exposed NS groups. However, in olfactory bulbs and the hypothalamus, exposure to O_3_ led to significantly higher levels of *Crhr2* expression in NS and SI but not CS respective to the matched air groups (Figure 2B).

Additionally, the expression of glucocorticoid (GRs; *Nr3c1*) and mineralocorticoid receptors (MRs; *Nr3c2*) was examined in all brain regions. Air-exposed SI animals had significantly lower *Nr3c1* expression relative to air-exposed NS in the hypothalamus but no other brain regions. Across nearly all selected brain regions, O_3_ exposure significantly decreased gene expression of *Nr3c1* regardless of the stress condition, except in the BNST of the CS group. In the hypothalamus of the SI groups, which already had depleted expression *of Nr3c1* at baseline, no effect of O_3_ was evident relative to air (Figure 2C). Additionally, MR in the brain had a much higher binding affinity for CORT compared to GR, despite *Nr3c2* expression being low. *Nr3c2* expression in air-exposed CS in BNST and SI in the hypothalamus was lower when compared to the matched NS group. *Nr3c2* expression was lower after O_3_ exposure relative to the matched air group in HIP (NS, CS and SI groups), HYP and OB (CS group) (Figure 2D). In the BNST, animals that underwent SI demonstrated higher expression of *Nr3c2* in response to O_3_ exposure (ANOVA Stress:Exposure F_(2,57)_ = 5.577; Pairwise t.ratio = −2.075; *p* < 0.05).

### 3.3. Gene Expression of Glucocorticoid-Associated Chaperone Proteins

**Figure 3:**
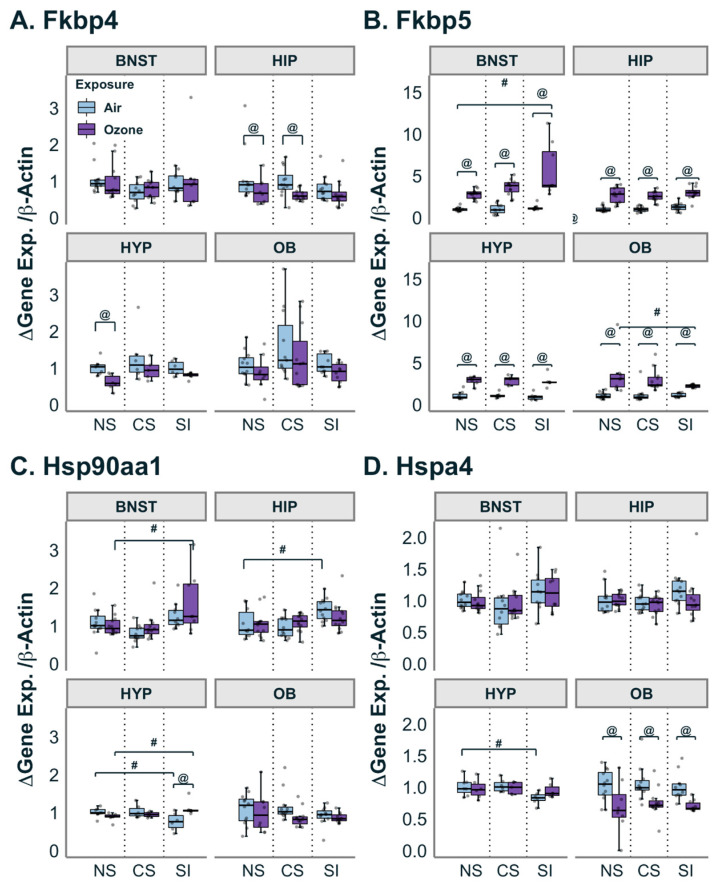
Effect of stressors on genes related to glucocorticoid-associated chaperones. (**A**): *Fkbp4* was significantly altered by exposure to O_3_ in the hippocampus (HIP) for the no stress (NS) and chronic stress (CS) groups and in the hypothalamus (HYP) for the NS group. (**B**): *Fkbp5* was significantly increased by O_3_ exposure in all brain regions. Also, the O_3_-induced increase in *Fkbp5* was higher than that in the NS group in the bed nucleus of stria terminalis (BNST) while it was lower in the olfactory bulb (OB). (**C**): *Hsp90aa1* expression was significantly higher in the BNST when exposed to O_3_ compared to the NS group. In the HYP, the social isolation (SI) group had higher baseline *Hsp90aa1* expression in the air group compared to NS. In the HYP, both exposure groups were significantly altered by SI compared to NS. (**D**): *Hspa4* was decreased by O_3_ exposure in the OB for all three stressor groups. In the HYP, the air-exposed group in SI had lower expression than that of the NS. Data are represented by boxplots with a dot plot overlay to express the distribution of each data point where *n* = 5–12. @ represents a statistical difference (*p* < 0.05; see details in the text) between exposure groups within the same stress group. # represents a statistical difference (*p* < 0.05; see details in the text) between stress groups within the same exposure group.

The sensitivity and function of GRs in the brain are heavily influenced by chaperone proteins as an additional regulatory mechanism [50,51]. In this study, we observed small brain-region-specific inhibition of *Fkbp4* expression by O_3_. O_3_ exposure inhibited *Fkbp4* expression in the hypothalamus of NS and hippocampus of NS and CS groups (Figure 3A). However, *Fkbp5* gene expression was elevated in all brain regions as a response to O_3_ exposure with a slightly exacerbated O_3_ effect in SI of BNST and slightly dampened O_3_ effect in SI of OB (Figure 3B).

Additionally, heat shock protein (HSP) gene expression was also assayed across all brain regions (Figure 3C,D). Two-way ANOVA revealed a significant effect of SI on the expression of *Hsp90aa1* in the BNST when compared to NS within the O_3_-exposed group, and likewise, a significant effect of SI in the hippocampus within air-exposed groups (F_(2,57)_ = 7.554, *p* < 0.005 and F_(2,64)_ = 6.487, *p* < 0.005; respectively). In the hypothalamus, the expression of *Hsp90aa1* was lower in air-exposed SI relative to air-exposed NS, but after O_3_ exposure in SI, its expression increased relative to air-exposed SI (F_(2,30)_ = 8.14; Pairwise t.ratio = −2.732 (air NS: air SI), 2.508 (O_3_ NS: O_3_ SI); *p* < 0.05) (Figure 3C). The gene *Hspa4*, which encodes for HSP70, showed no apparent expression changes upon either stress or O_3_ exposure except for in hypothalamus expression, which was lower in the SI air group relative to the NS air group. Interestingly, in the olfactory bulbs, *Hspa4* expression was decreased across all groups in response to O_3_ exposure (Figure 3D).

### 3.4. Gene Expression for Bdnf, Endocannabinoid Receptor (Cnr1) and Tyrosine Hydroxylase (Th)

**Figure 4:**
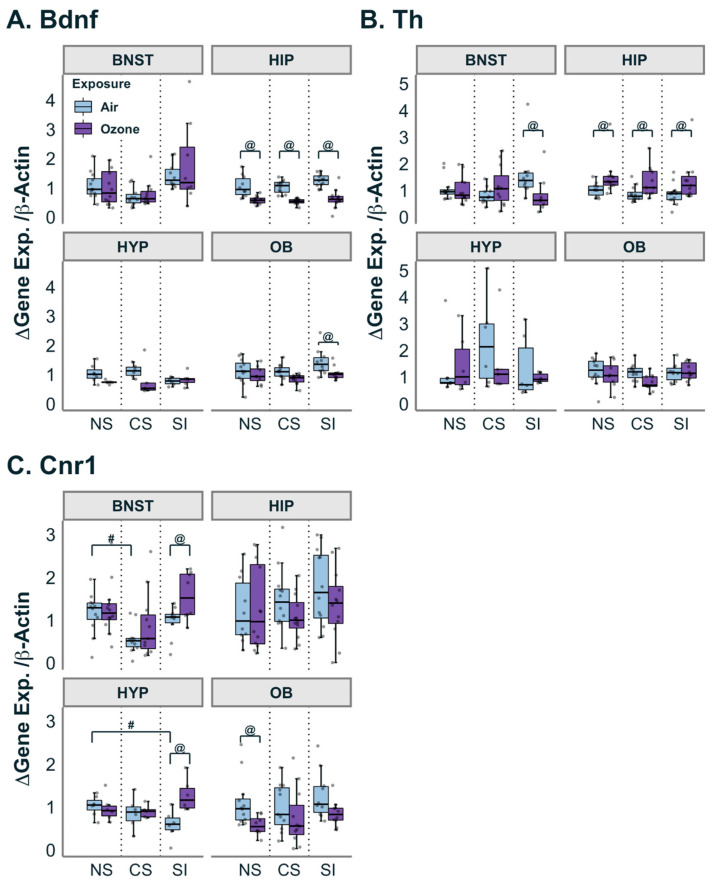
Effect of stressors on genes related to neurotrophic, neurotransmitter and cannabinoid factors. (**A**): O_3_ exposure significantly decreased *Bdnf* expression in the hippocampus (HIP) region for all three stress groups (NS = no stress controls; CS = chronic stress group; SI = social isolation group) and in the SI group in the olfactory bulb (OB). (**B**): *Th* expression was increased across all stress groups in response to O_3_ exposure in the hippocampus (HIP) but decreased in the SI within the bed nucleus of the stria terminalis (BNST). (**C**): *Cnr1* expression was increased by O_3_ exposure in the SI group within the BNST and hypothalamus (HYP) and decreased by O_3_ in the NS group within the OB. In the BNST, the baseline air-exposed group in the CS was lower than that of the NS. A lower baseline was also found in the SI of the HYP. Data are represented by boxplots with a dot plot overlay to express the distribution of each datapoint where *n* = 5–12. @ represents a statistical difference (*p* < 0.05; see details in the text) between exposure groups within the same stress group. # represents a statistical difference (*p* < 0.05; see details in the text) between stress groups within the same exposure.

While BDNF is ubiquitous across brain regions, some of the highest expression levels are found in the hippocampus [52,53]. In this study, there was a significant effect of O_3_ exposure on expression of *Bdnf* in the hippocampus, in that its expression was reduced regardless of the stress condition (Figure 4A). *Bdnf* expression was also decreased (*p* < 0.05) in the olfactory bulbs of the O_3_-exposed SI group relative to the air-exposed SI group. Additionally, within the hippocampus, the gene encoding for tyrosine hydroxylase, *Th*, was increased across all groups in response to O_3_ exposure (Figure 4B). This O_3_ effect was not apparent in other brain regions; instead, in BNST, O_3_ exposure led to inhibition of *Th* expression, but only in the SI group (Stress:Exposure F_(2,57)_ = 3.53; Pairwise t.ratio = 2.468 (SI air; SI O_3_; *p* < 0.05). The gene *Cnr1* encodes for cannabinoid receptor 1, the primary receptor for endocannabinoids. Within the BNST, there was a significant effect of both CS in air-exposed rats (Figure 4C; F_(2,57)_ = 5.704; *p* < 0.05) and O_3_ exposure (F_(1,57)_= 5.239; *p* < 0.05) in SI on *Cnr1* expression. In BNST, CS in air-exposed animals but not SI had significantly lower expression when compared to NS. O_3_ exposure relative to matching air, however, slightly increased expression of *Cnr1* in BNST, but only in the SI group. In the hypothalamus, a two-way ANOVA revealed a small but significant interaction between O_3_ exposure and SI (F_(2,30)_ = 5.207; *p* < 0.05), where SI in air-exposed animals had lower expression when compared to the air-exposed NS group; however, O_3_ exposure increased expression of *Cnr1* in the SI group when compared to the SI air group. Interestingly, in the olfactory bulbs, *Cnr1* expression was lower in the O_3_-exposed NS group relative to the air-exposed NS group (Figure 4C; F_(2,56)_ = 8.769; *p* < 0.005).

### 3.5. Metabolomic Analysis of Rat Serum Following Air or O_3_ Exposure in NS and SI

To gain more context for the brain-region-specific gene expression changes observed, serum metabolomics was conducted in the NS and SI groups since SI produced more changes when compared to CS relative to NS. Following O_3_ exposure, multiple glycerol, fatty acid and lipid pathways were significantly modified (Table 3).

Consistent with our previous findings [48,54], O_3_ alone, regardless of stress condition, was a potent modulator of pathways involving lipolysis, resulting in increases in free fatty acids and mono- as well as diacylglycerols. Interestingly, diacylglycerol pathways were affected by both acute O_3_ exposure and long-term SI, with the former inducing an increase and the latter a modest decrease in metabolite content. Sphingomyelins (SPs) were changed in the serum due to SI and O_3_ exposure independently. There was a significant O_3_ effect in NS and SI that caused an increased abundance of multiple SPs, and SI caused a concordant significant increase in these compounds when compared to the NS group within each exposure condition. Somewhat surprisingly, there were very few interactions between SI and O_3_ in the serum metabolomic analysis. The pathways that did show a significant interaction included fatty acid metabolism (dicarboxylate and acylcarnitine), mevalonate and lipid metabolism. While O_3_ caused a significant increase in these metabolites, prior sub-chronic SI slightly, but in some cases significantly, mitigated these effects.

## 4. Discussion

A single exposure to the air pollutant O_3_ activates the HPA and SAM axes [49]. We investigated whether underlying prolonged CS or SI could modify the sensitivity to O_3_-induced glucocorticoid and catecholamines release and, as a result, the circulating metabolome through transcriptional changes in their signaling markers in a brain-region-specific manner. Here, we demonstrate that O_3_-induced increases in circulating corticosterone and lymphopenia were not influenced by preexistent CS or SI; however, circulating BDNF was depleted in both stress conditions regardless of O_3_ exposure. SI in air-exposed animals dampened hypothalamic expression of glucocorticoid receptors and heat-shock proteins, while CS had smaller effects only in BNST (Table 3). O_3_ exposure was associated with widespread gene expression effects in all brain regions. O_3_ exposure changed markers of glucocorticoid signaling, which involved increased expression of the transcriptionally regulated glucocorticoid receptor scaffolding protein encoded by *Fkbp5* and inhibition of glucocorticoid receptor *Nr3c1*, generally in all brain regions. O_3_ also caused region- and stressor-specific inhibition of mineralocorticoid receptor *Nr3c2* expression. The expression of *Crhr1*, urocortin receptor 1 involved in inducing activation of the HPA axis, was neither changed by O_3_ nor stressors, but *Crhr2* expression was increased in the hypothalamus and olfactory bulbs of O_3_-exposed NS and SI animals. *Th* expression was inhibited only in the SI rats exposed to O_3_ in BNST, while it was increased in the hippocampus of all O_3_-exposed animals. Acute O_3_ exposure inhibited hippocampal *Bdnf* transcription regardless of stress condition. Global serum metabolomic assessment indicated increases in sphingomyelins in air-exposed SI and also by O_3_ in the NS and SI groups. However, we found moderate dampening of O_3_ effects on various sphingomyelins and other fatty acids in the O_3_-exposed SI group when compared to NS. Thus, the data support a small to moderate influence of SI on O_3_-induced changes in mRNA expression of genes involved in catecholaminergic and glucocorticoid signaling. These changes are brain-region-specific and suggest complex central integration of the acute O_3_-induced stressor response in rats with preexistent SI and CS. Further studies are needed to understand how these transcriptional changes are involved in modifying the normal stress response in individuals with psychosocial stress.

One of the ancillary objectives of this study was to obtain an insight into how preexistent long-term SI and CS might modify the neural catecholaminergic and glucocorticoid pathways activated by an acute O_3_ exposure used as a challenge stressor, with SI being an independent risk factor for chronic neural and systemic disease susceptibility. Our goal was to compare SI alone with CS employing a more severe stress condition by adding unpredicted daily mild stressors in the animals that were also socially isolated [34]. Unexpectedly, as recently published [34], the data demonstrated that many of the pulmonary and systemic effects observed in the air- and O_3_-exposed SI groups were smaller in the CS [22]. Thus, we believe that the CS paradigm including periodic and daily handling of animals for mild stress maneuvers and assessment of physiological responses likely nullified the effects of social isolation. The data on brain gene expression, in general, reflect this pattern. Therefore, metabolomic assessment was only performed for the NS and SI groups.

The focus on assessing the systemic stress response and transcriptional changes in glucocorticoid and catecholaminergic signaling markers in different brain regions was to determine if there are brain-region-specific alterations due to SI and CS that modify the ability of O_3_ to produce a stress response. Inability to produce a stress response due to underlying psychosocial stress has been considered a risk factor for exacerbation of air pollution health outcomes [11]. Our prior studies have shown that hypothalamic [20] and peripheral effects of acute O_3_ exposure are modulated by circulating adrenal-derived stress hormones, corticosterone and epinephrine [27], and that in the absence of circulating stress hormones, O_3_ effects are diminished [55]. Furthermore, adaptation following repeated O_3_ exposure was linked with the lack of transcriptional activation of corticosteroid synthesis enzymes within adrenals [56]. Thus, we presumed that preexistent SI and CS would impair acute O_3_-induced neuroendocrine stress mechanisms and focused on brain-region-specific transcriptional responses of receptors and chaperone proteins involved in glucocorticoid and catecholamine actions.

### 4.1. Preexistent Stressors and O_3_-Induced Brain-Region-Specific Transcriptional Response

The release of corticotrophin-releasing hormone (CRH) in the hypothalamus initiates the activation of the HPA axis after receiving a stress signal. Acute O_3_ exposure induces the activation of the SAM and HPA axes in a dynamic manner [49]. In addition, circulating glucocorticoids have direct feedback control on CRH release [57]. Although *Crh1* expression was not altered by O_3_ or preexistent CS or SI, we found that O_3_ exposure increased *Crhr2* expression in the hypothalamus and olfactory bulbs, but only in NS and SI animals. *Crhr1* encoding the corticotropic-releasing hormone receptor, being the main contributor to the urocortin-mediated activation of CRH release, means it is likely that *Crhr2*, responsive to circulating mineralocorticoid changes in rats [58], might contribute to O_3_-induced HPA activation. CRH signaling has been shown to participate in shaping activity-dependent intraneuronal connections and plasticity [59]. Presumably the first point of direct contact (olfactory epithelium) to O_3_ upon inhalation [60], it is likely that in addition to sensory input through trigeminal sensory nerves, the olfactory nerve activation could affect CRH signaling.

The inhibitory influence of SI in air-exposed animals on transcription of both GR receptors (NR3C1 and NR3C2) and heat shock proteins involved in regulating GR activity in the hypothalamus (Table 4) might suggest the contribution of SI in dampening O_3_ effects of HPA activation via corticosterone-induced feedback inhibition, despite circulating corticosterone in SI being non-significant relative to NS. The lack of a SI effect on O_3_-induced corticosterone increase might underlie the ultradian fluctuations and the altered dynamic nature of HPA activation [61]. The generalized inhibition of *Nr3c1*, the predominant GR gene after O_3_ exposure in all brain regions regardless of stress condition, could also be the result of increased circulating corticosterone. Although the mechanism by which long-term SI might inhibit expression of *Nr3c1* cannot be ascertained, the promoter region of *Nr3c1* has been shown to have binding sites for other transcription factors that provide fine-tuning of receptor expression in order to regulate glucocorticoid activity in a brain-region-specific manner [62].

Under basal conditions, the glucocorticoid receptor (encoded by *Nr3c1*) is bound to FKBP5 along with other proteins, forming a heterocomplex in the cytoplasm. When corticosterone binds to the GR receptor, FKBP5 is replaced by FKPB4 and thus allows the complex to translocate to the nucleus. Along with decreased *NR3C1*, increases in the FKBP5 that inhibits translocation of the GR complex to the nucleus are a part of the negative feedback loop that deactivates the HPA axis, thus regulating the longevity of the stress response at the central level [63]. O_3_-induced activation of transcriptionally regulated *Fkbp5* across all brain regions, regardless of stress condition, suggests that this effect is mediated by increased levels of circulating glucocorticoids and might serve to inhibit the stress response in order to avoid overt stress-mediated physiological responses as one of the regulatory mechanisms not influenced by SI or CS in the current experimental setting. We have recently reported that adaptation, despite continued O_3_ exposure, is linked to reduced stress-mediated glucocorticoid synthesis in the adrenals [56], thus suggesting that the *Fkbp5* gene might be involved in modulating reduced HPA stimulation through increased binding to GR and inhibiting its translocation to the nucleus and thereby its transcriptional activity after O_3_ exposure. It is noteworthy that in the BNST, SI animals had the highest O_3_-induced increase in *Fkbp5*, suggesting an interactive effect of O_3_ and SI on stress mechanisms. The impairment of *Fkbp5* functionality is associated with a variety of psychosocial stress conditions [64]; however, no specific insights could be obtained in regards to SI with current preliminary assessment of transcriptional changes.

Hsp70 is part of the GR heterocomplex that aids in the folding of the nascent GR protein. Although Hsp70 is extensively used for various other proteins’ folding, it is plausible that the O_3_-induced decrease in its expression in olfactory bulbs together with decreased *Nr3c1* (GR) expression could be involved in glucocorticoid activity regulation. Across various brain regions, differential stress and O_3_ impact on expression of *Hsp90aa1*, which codes for another GR chaperone protein HSP90, thus leading to the complexity of GR regulation under different stress conditions. Since the expression of these chaperones and other regulatory proteins was analyzed in dissected whole brain regions, it is difficult to delineate if these coordinated gene expression changes are specific to given neural bundles. In future studies, identifying the location and cell-type composition of these neuronal circuits will be important to better understand the impact of SI and CS on O_3_-induced stress dynamics.

The O_3_-induced changes in hippocampal *Bdnf* and *Th* gene expression are noteworthy. *Bdnf* is a critical neurotrophic factor that promotes neuronal survival and has the highest expression in the hippocampus, contributing to circulating BDNF [65]. It has been demonstrated that both acute and chronic restraint stress diminish *Bdnf* mRNA in the hippocampus [66]. In the present study, although CS and SI reduced circulating BDNF protein, we did not observe significant changes in *Bdnf* expression in any brain region, which might be attributed to the intensity and longevity of stress. Changes in BDNF levels and expression have been reported in humans and experimental studies [67,68]. Interestingly, acute O_3_ exposure, which induces a robust stress response, decreased *Bdnf* mRNA in the hippocampus regardless of underlying chronic stress, which might indicate the involvement of the hippocampus and could be linked to chronicity of the stress response since circulating BDNF protein levels were decreased by SI and SC regardless of O_3_. Hippocampal *Bdnf* expression has been shown to play a critical role in resilience to chronic stress, and reduction in hippocampal *Bdnf* expression induces prolonged elevations in corticosterone secretion [69]. In cultured cortical cells, *Bdnf* can provoke increased *Th* gene expression via specific Ca^2+^ channels [70]. Repeated restraint in rats has been shown to increase tyrosine hydroxylase immunoreactivity and decrease BDNF levels in the hippocampus [71]. This suggests a potential link between *Bdnf* and the catecholaminergic system within the hippocampus. In this study, acute O_3_-induced inhibition of hippocampal *Bdnf* expression, together with long-term CS- and SI-induced decreases in circulating BDNF protein, suggests its potential involvement in observed peripheral effects of SI and CS on O_3_-induced systemic effects [34]. Hippocampal *Th* expression, which was increased after O_3_ exposure (together with decreased *Bdnf* expression), and circulating BDNF levels have been linked to systemic inflammation [72]. The involvement of BDNF will need to be further investigated with spatiotemporal assessment. Nevertheless, this hippocampus-specific effect might suggest its contribution in O_3_ and the SI stress dynamic.

### 4.2. Systemic Metabolic Impacts of SI and O_3_ through Neuroendocrine System

Serum metabolomic analysis provided insights into some of the shared systemic responses between acute O_3_ exposure and sub-chronic SI. As we have demonstrated previously, O_3_ at 0.8 ppm for 4 h caused major changes in most pathways, with an especially notable signal for those involved in complex lipid metabolism [42,48,54]. These O_3_-induced changes were observed regardless of SI. The acute O_3_-induced metabolic changes are consistent with neuroendocrine-mediated increases in circulating epinephrine and corticosterone [49] because both of these hormones are known to mediate metabolic changes in adipose tissue, liver and muscle and regulate glucose-mediated insulin release [26,49,73]. Mono- and diacylglycerol metabolites, as well as lysophospholipids and free fatty acids, were significantly increased in response to O_3_ among both the NS and SI groups, with slightly reduced O_3_ effects in the SI group, suggesting that the prolonged SI may have dampened acute O_3_-induced stress and resulting lipolysis. Sphingomyelin metabolites were significantly increased in response to SI among both the air- and O_3_-exposed groups. However, the interaction between O_3_ and SI was not statistically significant, suggesting that the long-term SI acted similarly to acute O_3_ and did not prime these animals for an exacerbated sphingomyelin response. Signatures related to sphingomyelin changes, especially from the sub-chronic SI, may be related to the depression phenotype [74].

The primary interaction observed between SI and O_3_ was related to lipid catabolism. Fatty acid dicarboxylates increased dramatically from O_3_ exposure in NS; however, this increase was slightly dampened in SI animals exposed to O_3_. Fatty acid dicarboxylates, ketone bodies and branched-chain amino acids increase when fatty acids are too high in tissues, and the results are consistent with our previously published data showing dampened O_3_-induced increases in serum branched-chain amino acids in SI animals relative to NS [34]. The serum sphingolipid modifications in air- and O_3_-exposed SI are associated with SI-induced dampened transcription of GR receptor and chaperone protein expression in the hypothalamus; however, specific linkages will need to be further examined.

### 4.3. Study Limitations

There are a number of limitations in this study that warrant mention. The changes in brain regions were examined at only the transcriptional level and correlated with circulating stress hormones, while protein levels were not assessed for given gene markers. These initial findings on gene expression changes allow us to postulate the potential interaction between chemical and non-chemical stressors involving glucocorticoids and catecholamines, and to identify the brain regions involved. Focused interventional studies are needed to identify the mechanisms by which catecholamines and glucocorticoids, under different psychosocial stress conditions, modify stress responses. Moreover, the stressor paradigm used for mild CS for male adolescent WKY rats in the present study, although including single housing with a lack of enrichment, could have negated the influence of SI through frequent daily handling and movement of rats with mild stress. Extended stressor application could have provided stronger stress-related changes. We examined the effects of O_3_ only in male rats, based on our general observation that for this strain of rat, females are less sensitive to inhaled pollutant-induced lung injury and inflammation [75]; however, it is important to consider sex differences in the involvement of neuroendocrine responses in relation to stress mechanisms. The stress response to O_3_ is dynamic and self-regulatory [49]. One-time assessment of biological endpoints is likely to miss important differences especially with acute stressor application.

### 4.4. Conclusions

We have recently shown that CS- and SI-induced systemic inflammation, hypercholesterolemia, and reductions in circulating pituitary hormones involved in thyroid and gonadal axes were exacerbated by acute O_3_ exposure [34]. Through further examination of stress-responsive brain regions and systemic stress markers, we wanted to gain an understanding of how underlying CS and SI might modify O_3_-induced neural catecholaminergic and glucocorticoid signaling by assessing transcriptional levels of respective mediators. Overall, we show that CS or SI did not change circulating adrenal-derived stress hormones but reduced BDNF levels. These changes were associated with dampened transcription of hypothalamic glucocorticoid signaling markers in SI. O_3_ exposure was associated with increases in circulating epinephrine and corticosterone regardless of stress condition. Increases in circulating adrenal hormones were associated with brain-region-specific changes in mRNA expression of a number of catecholaminergic and glucocorticoid-signaling proteins involved in regulation of plasticity of a stress response. O_3_ exposure inhibited *Bdnf* and increased *Th* transcription primarily in the hippocampus, with no impact of prior stress condition. These changes with higher levels of circulating epinephrine in all O_3_-exposed rats regardless of stress condition suggest their likely contribution in modulating hippocampal stress regulatory mechanisms. Serum metabolomic analysis indicated increases in sphingomyelins by each stressor, O_3_ and SI, with SI slightly reducing O_3_-induced changes. The transcriptional changes induced by SI and/or O_3_ were brain-region- and stressor-specific but indicated a potential role of glucocorticoid mechanisms associated with increased levels of circulating glucocorticoids. In light of consistent linkage between exposure to air pollutants and psychosocial stressors, it will become critical to understand how chemical and non-chemical stressors interact to exacerbate neural and peripheral diseases through neuroendocrine impairment [76].

## Figures and Tables

**Table 1:** List of stressors in this study.

Stressor	Description
Restraint	Rats are placed in size-appropriate nose-only inhalation exposure tubes that are arranged on a rack for 1 h. To ensure the rat is immobilized and unable to turn around in the tube, foam pieces are added to decrease the length of the tube where needed.
Tilted cage	Rats (1/cage) are placed in cages tilted at 45° for 1 h. The cages have a wire mesh bottom for added grip but are without bedding, food or water.
Shaking	Rats are placed in cages with dividers that separate the cages into 4 equal quadrants (1 rat/quadrant). Three cages are placed on a modified orbital plate shaker set at 100 rpm for 1 h.
Noise	Intermittent white noise of 85 dB is broadcast from speakers located above each individual cage. A timer is set to vary on–off times of the noise, from 5 to 25 min with 5 to 45 min between noise bursts for a total of 6 h.
Predator odor	Rats are exposed to a predator odor (2,5-dihydro-2,4,5-trimethylthiazoline, a chemical isolated from fox urine, which is extensively used for triggering defensive behaviors in rodents) for 1 h. Since this chemical is volatile, a small amount is placed on gauze pads in an open Petri dish placed in the middle of the animal room. The room is maintained at negative pressure to avoid spreading of odor in other areas.

**Table 2:** List of PCR primers used in this study. * Indicates housekeeper genes.

Gene Symbol	Accession Number	Forward Primer Sequence	Forward Tm	Reverse Primer Sequence	Reverse Tm	Product Length	Efficiency
Actb	NM_031144.3	GTGTGGATTGGTGGCTCTATC	58.43	AACGCAGCTCAGTAACAGTC	58.22	137	96.184
* Gapdh	NM_017008.4	ACTCCCATTCTTCCACCTTTG	57.84	GTCCAGGGTTTCTTACTCCTTG	58.32	155	109.310
* Rpl13A	XM_017589309.1	TACTCTGGAGGAGAAACGGAAG	58.91	ACCTACAGGAGCAGTGACTAAG	58.91	257	96.689
Fkbp4	NM_001191863.1	TCATCAAGAGAGAGGGTACAGG	58.36	TGGTTGCCACAGCAATATCC	58.53	183	103.394
Fkbp5	NM_001012174	CACCAGTAACAATGAAGAAAACCC	58.47	CCTCACTAGTCCCCACTCTT	57.76	116	108.288
Hsp90aa1	NM_175761.2	AAACAGCACTCCTGTCTTCC	57.74	GCCTAGTCTACTTCTTCCATGC	58.28	199	103.447
Hspa4	NM_153629.1	ACCACCTCAAGCAAAGAAGG	58.01	CCGTTCCTTCTCCAGTTTATCC	58.47	154	97.097
nr3c1	NM_012576.2	CCTTTGTTCTAAGCTAGGGAAGG	58.48	GTGGATGAGGATGGTTAGAATGG	58.61	127	96.072
nr3c2	NM_013131.1	GGCAAATCTCAACAACTCAAGG	58.09	TGAAGTGGCATAGCTGAAGG	57.59	142	105.798
Th	NM_012740	TCGGGCTATGTAAACAGAATGG	58.20	CTGGTAGGTTTGATCTTGGTAGG	58.23	158	96.901
Bdnf	NM_001270630.1	GGTCGATTAGGTGGCTTCATAG	58.35	CGGAAACAGAACGAACAGAAAC	58.40	160	98.044
Crhr1	NM_030999	GGTATACACTGACTACATCTACCAG	57.80	CAGCCTTCCTGTACTGAATGG	58.36	143	100.945
Crhr2	NM_022714	CAGATTGTGTTCATCTACTTCAACTC	58.26	GTGCCACCGCTTTCTCA	57.46	117	106.529
Cnr1	NM_012784.5	GGCATCAGGGTTATCTACTTCC	57.99	ACAGCTTTGGAGACATCTGG	57.51	263	106.874

**Table 3:** Summary of modulated metabolic pathways from serum in animals exposed to O_3_ along with different stressors.

	ANOVA Global			Two-Way ANOVA Contrasts			
	**SI**	**O_3_**	**Interaction**	**NH**	**SI**	**SI:NH**	
**Pathway**				O_3_:Air	O_3_:Air	Air:Air	O_3_:O_3_
**Glycerolipid Metabolism**							
*glycerol*	-	Y	-	1.37 (↑)	1.53 (↑)	0.85	0.94
*glycerophosphoglycerol*	-	Y	-	1.27 (↑)	1.62 (↑)	0.81	1.03
**Monoacylglycerols**							
*1-oleoylglycerol (18:1)*	-	Y	-	1.26	1.76 (↑)	0.70	0.97
*1-linoleoylglycerol (18:2)*	-	Y	-	1.70 (↑)	1.69 (↑)	0.87	0.86
*1-linolenoylglycerol (18:3)*	-	Y	-	2.03 (↑)	1.75 (↑)	0.84	0.72
**Diacylglycerols**							
*palmitoyl-oleoyl-glycerol (16:0/18:1)*	Y	Y	-	1.57 (↑)	1.64 (↑)	0.80	0.84
*palmitoyl-linoleoyl-glycerol (16:0/18:2)*	Y	Y	-	1.56 (↑)	1.63 (↑)	0.76 (↓)	0.79 (↓)
*palmitoyl-arachidonoyl-glycerol (16:0/20:4)*	-	Y	-	2.04 (↑)	2.61 (↑)	0.81	1.04
*linoleoyl-linoleoyl-glycerol (18:2/18:2)*	Y	Y	-	1.45 (↑)	1.23	0.74 (↓)	0.63 (↓)
**Sphingomyelins (SPs)**							
*palmitoyl sphingomyelin (d18:1/16:0)*	Y	Y	-	1.11 (↑)	1.13 (↑)	1.07	1.09 (↑)
*stearoyl sphingomyelin (d18:1/18:0)*	Y	Y	-	1.14 (↑)	1.14 (↑)	1.15 (↑)	1.15 (↑)
*behenoyl sphingomyelin (d18:1/22:0)*	Y	Y	-	1.24 (↑)	1.18 (↑)	1.17 (↑)	1.11 (↑)
*tricosanoyl sphingomyelin (d18:1/23:0)*	Y	Y	-	1.25 (↑)	1.15 (↑)	1.15 (↑)	1.06
*lignoceroyl sphingomyelin (d18:1/24:0)*	Y	Y	-	1.24 (↑)	1.22 (↑)	1.11 (↑)	1.09 (↑)
*SP (d18:2/18:1)*	Y	Y	-	1.71 (↑)	1.45 (↑)	1.33 (↑)	1.13
*SP (d18:2/23:1)*	Y	Y	-	1.14 (↑)	1.12	1.14 (↑)	1.12
*SP (d18:2/24:2)*	Y	Y	-	1.15 (↑)	1.09	1.13 (↑)	1.07
*SP (d18:1/14:0, d16:1/16:0)*	Y	Y	-	1.13 (↑)	1.16 (↑)	1.13 (↑)	1.16 (↑)
*SP (d18:1/19:0, d19:1/18:0)*	Y	Y	-	1.50 (↑)	1.43 (↑)	1.23 (↑)	1.18 (↑)
*SP (d18:1/20:0, d16:1/22:0)*	Y	Y	-	1.28 (↑)	1.25 (↑)	1.16 (↑)	1.13 (↑)
*SP (d18:1/20:1, d18:2/20:0)*	Y	Y	-	1.15 (↑)	1.17 (↑)	1.17 (↑)	1.19 (↑)
*SP (d18:1/20:2, d18:2/20:1, d16:1/22:2)*	Y	Y	-	2.35 (↑)	2.40 (↑)	1.58	1.61 (↑)
**Fatty Acid Metabolism, Dicarboxylate**							
*3-hydroxyadipate*	Y	Y	Y	2.52 (↑)	1.44 (↑)	0.98	0.56 (↓)
*pimelate (C7-DC)*	-	-	Y	1.43 (↑)	0.90	1.09	0.69 (↓)
*tetradecanedioate (C14-DC)*	Y	Y	Y	1.49 (↑)	1.13	0.99	0.75 (↓)
**Fatty Acid Metabolism, Acylcarnitine**							
*oleoylcarnitine (C18:1)*	Y	Y	Y	1.45 (↑)	1.23 (↑)	0.97	0.83 (↓)
*(R)-3-hydroxybutyrylcarnitine*	-	Y	Y	2.51 (↑)	1.67 (↑)	1.18	0.79 (↓)
*(S)-3-hydroxybutyrylcarnitine*	-	Y	Y	1.29 (↑)	1.04	1.06	0.85 (↓)
**Mevalonate Metabolism**							
*3-hydroxy-3-methylglutarate*	Y	Y	Y	1.34 (↑)	1.03	0.97	0.75 (↓)
*mevalonolactone*	Y	-	Y	1.29	0.69	0.87	0.46 (↓)
**Lipid Metabolism**							
*nicotinamide*	-	Y	Y	1.87 (↑)	1.31 (↑)	1.06	0.74 (↓)
*carnitine*	-	Y	Y	0.77 (↓)	0.88 (↓)	0.99	1.13
*3-hydroxybutyrate (BHBA)*	Y	Y	-	1.38 (↑)	1.16	0.97	0.82 (↓)
*4-hydroxybutyrate (GHB)*	Y	Y	Y	1.59 (↑)	1.10	1.03	0.72 (↓)

↑ Indicates an increased fold change (red) and ↓ indicates a decrease in fold change (blue). No change is denoted as black. Social isolation (SI), ozone (O_3_), normal housing (NH).

**Table 4:** Summary of O_3_ and/or stress effects on circulating neuroendocrine hormones and brain-region-specific gene expression.

Serum/Brain Region	Marker	CS Effect in Air	SI Effect in Air	O_3_ Effect in NS	O_3_ Effect in CS	O_3_ Effect in SI
Serum	Epinephrine	-	-	↑	↑	↑
	Nor-epinephrine	-	-	-	-	-
	ACTH	-	-	-	-	-
	Corticosterone	-	-	↑	↑	↑
	Lymphocytes	-	-	↓	↓	↓
	BDNF	↓	↓	-	↓	↓
*BNST*	*Fkbp4*	-	-	-	-	-
	*Fkbp5*	-	-	↑	↑	↑
	*Hsp90aa1*	*-*	*-*	*-*	*-*	↑
	*Hspa4*	*-*	*-*	*-*	*-*	*-*
	*Nr3c1*	*-*	*-*	↓	*-*	↓
	*Nr3c2*	↓	*-*	*-*	↓	*-*
	*Th*	*-*	*-*	*-*	*-*	↓
	*Bdnf*	*-*	*-*	*-*	*-*	*-*
	*Crhr1*	*-*	*-*	*-*	*-*	*-*
	*Crhr2*	*-*	*-*	*-*	*-*	*-*
	*Cnr1*	↓	*-*	*-*	*-*	↓
*Hippocampus*	*Fkbp4*	*-*	*-*	↓	↓	*-*
	*Fkbp5*	*-*	*-*	↑	↑	↑
	*Hsp90aa1*	*-*	↑	*-*	*-*	-
	*Hspa4*	*-*	*-*	*-*	*-*	*-*
	*Nr3c1*	*-*	*-*	↓	↓	↓
	*Nr3c2*	*-*	*-*	↓	↓	↓
	*Th*	*-*	*-*	↑	↑	↑
	*Bdnf*	*-*	*-*	↓	↓	↓
	*Crhr1*	*-*	*-*	*-*	*-*	*-*
	*Crhr2*	*-*	*-*	*-*	*-*	*-*
	*Cnr1*	*-*	*-*	*-*	*-*	*-*
*Hypothalamus*	*Fkbp4*	*-*	*-*	↓	*-*	*-*
	*Fkbp5*	*-*	*-*	↑	↑	↑
	*Hsp90aa1*	*-*	↓	*-*	*-*	↑
	*Hspa4*	*-*	↓	*-*	*-*	*-*
	*Nr3c1*	*-*	↓	↓	↓	↓
	*Nr3c2*	*-*	↓	*-*	↓	↓
	*Th*	*-*	*-*	*-*	*-*	*-*
	*Bdnf*	*-*	*-*	*-*	*-*	*-*
	*Crhr1*	*-*	*-*	*-*	*-*	*-*
	*Crhr2*	*-*	*-*	↑	*-*	↑
	*Cnr1*	*-*	*-*	*-*	*-*	*-*
*Olfactory bulb*	*Fkbp4*	*-*	*-*	*-*	*-*	*-*
	*Fkbp5*	*-*	*-*	↑	↑	↑
	*Hsp90aa1*	*-*	*-*	*-*	*-*	*-*
	*Hspa4*	*-*	*-*	↓	↓	↓
	*Nr3c1*	*-*	*-*	↓	↓	↓
	*Nr3c2*	*-*	*-*	*-*	↓	*-*
	*Th*	*-*	*-*	*-*	*-*	*-*
	*Bdnf*	*-*	*-*	*-*	*-*	↓
	*Crhr1*	*-*	*-*	*-*	*-*	*-*
	*Crhr2*	*-*	*-*	↑	*-*	↑
	*Cnr1*	*-*	*-*	↓	*-*	*-*

An increase is indicated by ↑ and a decrease by ↓ for serum levels of markers and relative mRNA changes in different brain regions. BNST, bed nucleus of stria terminalis; ACTH, adrenocorticotropic hormone, BDNF, brain-derived natriuretic factor.

## Data Availability

Data is contained within the article.
